# Adaptive responses to air pollution in human dermal fibroblasts and their potential roles in aging

**DOI:** 10.1096/fba.2021-00056

**Published:** 2021-07-17

**Authors:** Wil J. Reynolds, Amy Bowman, Peter S. Hanson, Adam Critchley, Ben Griffiths, Bhaven Chavan, Mark A. Birch‐Machin

**Affiliations:** ^1^ Dermatological Sciences, Translational and Clinical Research Institute Newcastle University Newcastle upon Tyne UK; ^2^ Mental Health Dementia and Neurodegeneration, Translational and Clinical Research Institute Newcastle University Newcastle upon Tyne UK; ^3^ Royal Victoria Infirmary Newcastle upon Tyne UK; ^4^ Croda Europe Ltd Snaith UK

**Keywords:** antioxidant, mitochondria, oxidative stress, pollution, skin

## Abstract

The damaging effects of air pollution on the skin are becoming increasingly researched and the outcomes of this research are now a major influence in the selection and development of protective ingredients for skincare formulations. However, extensive research has not yet been conducted into the specific cellular defense systems that are being affected after exposure to such pollutants. Research investigating the affected systems is integral to the development of suitable interventions that are capable of augmenting the systems most impacted by air pollutant exposure. The following studies involved exposing primary human dermal fibroblasts to different concentrations of particulate matter and analyzing its effects on mitochondrial complex activity, nuclear factor erythroid 2‐related factor 2 localization using immunocytochemistry and protein expression of electron transport chain complex proteins, sirtuin‐1 (SIRT1), and peroxisome proliferator‐activated receptor gamma coactivator‐1α (PGC‐1α) using western blotting. Particulate matter‐induced alterations in both mitochondrial complex protein and activity, indicating oxidative stress, which was also complimented by increased expression of antioxidant proteins GSTP1/2 and SOD2. Particulate matter also seemed to modify expression of the proteins SIRT1 and PGC‐1α which are heavily involved in the regulation of mitochondrial biogenesis and energy metabolism. Given the reported results indicating that particulate matter induces damage through oxidative stress and has a profound effect on mitochondrial homeostasis, interventions involving targeted mitochondrial antioxidants may help to minimize the damaging downstream effects of pollutant‐induced oxidative stress originating from the mitochondria.

**Abbreviations**
ARE: antioxidant response element
ATP: adenosine triphosphate
COXVA: cytochrome C oxidase subunit 5A
DMSO: dimethyl sulfoxide
ETC: electron transport chain
GST: glutathione s‐transferase
KEAP1: Kelch‐like ECH‐associated protein 1
MEK: mitogen‐activated protein kinases (MAPK)/extracellular signal‐regulated kinases (ERK)
MMP: matrix metalloproteinase
mtDNA: mitochondrial DNA
nDNA: nuclear DNA
NDUFA9: NADH:ubiquinone oxidoreductase subunit A9
NQO1: NAD(P)H quinone dehydrogenase‐1
NRF‐1: nuclear respiratory factor‐1
Nrf2: nuclear factor erythroid 2–related factor 2
NRF‐2: nuclear respiratory factor‐2
PGC‐1α: peroxisome proliferator‐activated receptor gamma coactivator 1‐alpha
PM: particulate matter
ROS: reactive oxygen species
SDHA: succinate dehydrogenase complex flavoprotein subunit A
SIRT1: sirtuin‐1
SOD: superoxide dismutase
UCCR: ubiquinol‐cytochrome C reductase core protein I
UV: Ultraviolet radiation

## 1 INTRODUCTION

The mitochondria are responsible for producing the energy serving molecule adenosine triphosphate (ATP) via the action of oxidative phosphorylation, a process involving both the electron transport chain (ETC) and chemiosmosis. This molecule supplies the cell with the energy required to maintain normal cellular functions, this is substantiated by the fact that high‐energy demanding cells like hepatocytes have ~2500 mitochondria per cell, in contrast to only ~200 mitochondria per cell for low‐energy demanding cells such as dermal fibroblasts.^1^ In the process of generating ATP, electrons have been shown to leak from complexes I, II, and III, and subsequently can react with molecular oxygen to form superoxide reactive oxygen species (ROS).^2^ Cells can attenuate ROS effects by dismutation of superoxide to hydrogen peroxide via the mitochondrial antioxidant manganese superoxide dismutase (SOD2); however, this process is not completely efficient, with a percentage of hydrogen peroxide being converted to hydroxyl radicals via the Fenton reaction. Hydroxyl radicals are more potent at oxidizing cellular proteins, lipids, and nucleic acids, and are likely the culprits of the genetic and structural damage caused by oxidative damage.^3^ Once an excess of ROS accumulates, the cell's antioxidant capacity becomes overwhelmed in what is now commonly known as oxidative stress. This is thought to contribute to the pathophysiology of a number of diseases, as well as in the process of aging.^4^ Excess ROS cannot only be generated by the ETC but also be induced by extrinsic factors (such as UV and air pollution), where they can damage cellular components directly or indirectly by the activation of pathways such as MAPK/ERK (MEK) pathways.^5^ These pathways are responsible for inducing the transcription of both proinflammatory cytokines and matrix metalloproteinases (MMPs), and for this reason the roles extrinsic factors such as UV and air pollution play in premature skin aging and inflammatory skin conditions are being investigated.^6^

Mitochondrial ETC complex proteins are maintained mainly by nuclear DNA (nDNA) and imported into the mitochondria; however 13 are encoded by mitochondrial DNA (mtDNA) and are synthesized inside the mitochondria.^7^ Peroxisome proliferator‐activated receptor gamma coactivator‐1α (PGC‐1α) modulates transcription factors that are responsible for regulating these complex protein‐encoding genes and also for proteins involved in mtDNA transcription and regulation. Such transcription factors include nuclear respiratory factors 1 and 2 (NRF‐1 and NRF‐2), which both regulate nuclear genes that are directly involved in mitochondrial biogenesis and mtDNA maintenance.^7^ Induction of PGC‐1α occurs via a number of different mechanisms, however the two most pertaining to the mitochondria are deacetylation by sirtuin‐1 (SIRT1) activated as a consequence of an increasing cytosolic NAD+/NADH ratio, and phosphorylation by p38/JNK MEK activated as a consequence of oxidative stress.^8^, ^9^

Nuclear factor erythroid 2‐related factor 2 (Nrf2) is a ligand‐activated transcription factor, which under normal conditions is bound to cytoplasmic Kelch‐like ECH‐associated protein 1 (KEAP1) to avoid unnecessary over‐activation of antioxidant defenses. To combat the ROS produced from normal cellular homeostasis, a small proportion of Nrf2 dissociates and translocates to the nucleus so it can regulate transcription of defenses against oxidative stressors through the antioxidant response element. Such defenses include phase II metabolizing enzymes (e.g., NAD(P)H quinone oxidoreductase [NQO1] and glutathione‐s transferases [GSTs]), and antioxidant enzymes (e.g., SOD1, SOD2, and glutathione peroxidase).^10^ The phase II metabolizing enzymes conjugate harmful chemicals with charged species to make them less toxic and more easily excreted, GSTs for example, conjugate glutathione to detoxify electrophiles such as quinones, and any ROS they generate through redox cycling.^11^, ^12^ Antioxidant enzymes also protect against oxidative stress by neutralizing any excess ROS; however, continual cellular exposure to oxidative stressors can overwhelm these antioxidant defenses, leaving the cell open to increased oxidative damage.^13^

The prevailing theory of aging is the mitochondrial free‐radical theory of aging, which states that as we age our cellular functions start to decline, including the aforementioned defense systems against oxidative stress, leading to an increase in mitochondrial ROS that are free to damage cellular components.^14^ This theory also postulates that due to the increased ROS, the mtDNA that is localized in close proximity to the ETC and has limited protective histones/repair mechanisms could undergo oxidative DNA damage. This could then translate into the production of dysfunctional ETC subunits which may be less efficient at transferring electrons and cause further ROS leakage, augmenting the initial ROS‐induced damage.^15^ This “vicious cycle of aging” is exacerbated further if extrinsic stressors such as UV and pollutants are also contributing to ROS generation.

This study will investigate the effect of the air pollution component particulate matter on a number of adaptive mechanisms in primary dermal fibroblasts.

## 2 MATERIALS AND METHODS

### 2.1 Preparation of particulate matter

SRM 1649b aka “urban dust particulate matter” (PM) was purchased from the National Institute of Standards and Technology, and a master stock prepared at a concentration of 50 mg/ml in dimethyl sulfoxide (DMSO). Prior to the preparation of culture treatments, the master stock was sonicated in a sonic bath for 1 h at 25°C with regular vortexing to avoid any agglomeration of particles. After the addition of PM to fibroblast medium, experiments were performed within 1 h of stock preparation to limit any variability in solution composition.

### 2.2 Cell culture

Primary human dermal fibroblasts were isolated from human female breast tissue, obtained from the Royal Victoria Infirmary, Newcastle, UK, following standardized methods. Fibroblasts were routinely maintained in culture medium (Advanced DMEM medium supplemented with 10% (v/v) fetal bovine serum, 1% (v/v) Glutamax, and 100 U of Penicillin/Streptomycin) on gelatin coated 75‐cm^2^ flasks at 37°C with 5% CO_2_ within a humidified incubator. For exposure experiments fibroblasts were exposed to fresh medium with PM every 2 days.

### 2.3 Immunocytochemistry

After incubating with PM for 24 h, cells were washed with phosphate‐buffered saline (PBS), before fixing with 3.7% (w/v) paraformaldehyde and permeabilized with 70% (v/v) ethanol. After cells were washed with PBS and blocked with 5% normal goat serum in PBS, cells were incubated with Nrf2 (1/500; Cell Signaling Technology) in antibody incubation buffer (1% bovine serum albumin, 0.2% Triton‐X100 in PBS). After overnight incubation at 4°C, cells were washed with PBS, followed by a 1 h incubation of Alexa Fluor 594 IgG‐conjugated secondary antibody (ThermoFisher Scientific). Cells were once again washed before counterstaining with DAPI solution (300 nM) for 5 min at room temperature, washed with PBS, and mounted with Prolong Glass Antifade mountant. Cells were imaged using the Zeiss Axioplan 2 microscope (Zeiss). For quantification 200–300 cells were analyzed (around 25–30 fields of view) using Image J software (NIH), with the DAPI channel thresholded to provide a nuclear mask that allowed the measurement of nuclear Nrf2 protein. Integrated density (the product of mean gray value and area) was used as a measure for the total amount of protein in a given region of interest.

### 2.4 Western blotting

Cell lysates were prepared by scraping cells in native lysis buffer (1% v/v Triton‐X100, 1% 10X TBS, 0.27 M sucrose, 1X protease inhibitor cocktail), sonicating on ice using the SONICS Vibra‐Cell 505 sonication probe for 20 s. The lysates were then centrifuged for 20 min at 13,500 *g* at 4°C and the supernatant was removed and protein concentration determined using a Bradford assay. Twenty micrograms of protein were subjected to SDS‐PAGE using 4–12% Bis‐Tris gels (Invitrogen) before using the iBlot2 to electro‐transfer the proteins on to a nitrocellulose membrane. Membranes were blocked using Odyssey blocking buffer (Licor) for 1 h at room temperature before incubating the membranes with primary antibody in antibody incubation buffer (Odyssey buffer with 0.2% Tween‐20) overnight at 4°C. Primary antibodies used were ubiquinol cytochrome‐c reductase core protein 1 (UCCR; Abcam), cytochrome‐c oxidase subunit 5a (COXVA; Mitosciences), succinate dehydrogenase complex flavoprotein subunit‐A (SDHA; Abcam), NADH‐ubiquinone oxioreductase subunit‐A9 (NDUFA9; Mitosciences), SOD2 (Abcam), GSTP1/2 (Santa Cruz), SIRT1 (Santa Cruz), and phosphorylated PGC‐1α (R&D Systems). Following incubation membranes were washed with TBS + 0.2% Tween‐20 three times for 5 min, before incubating with IRDye 800CW secondary antibody (Licor) in antibody incubation buffer for 1 h at room temperature. Membranes were washed again, before incubation with glyceraldehyde‐3‐phosphate dehydrogenase (GAPDH) antibody conjugated to an Alexa Fluor 680 fluorophore (Santa Cruz) for 1 h at room temperature in antibody incubation buffer. Since whole cell lysate was used, GAPDH serves as a reliable loading control to account for any changes in cell number. Membranes were washed for a final time before imaging using the Licor Fc Dual Mode Imaging System.

### 2.5 Spectrophotometric analysis of mitochondrial complex activity

Photometric assays were conducted using a Cary 300 Bio UV‐Visible spectrophotometer (Varian Inc.), and results were visualized using Cary WinUV Kinetics Application (Varian Inc.). Cells were washed with PBS before detaching cells using trypsin‐ethylenediaminetetraacetic acid and collecting via centrifugation at 200 *g* for 5 min. The cell pellet was resuspended in complex buffer (25 mM potassium phosphate, 5 mM magnesium chloride in deionized water [dH_2_O], pH 7.2), before subjecting to three snap freeze–thaw cycles in liquid nitrogen to burst cell membranes. Citrate synthase activity was used to determine mitochondrial number in each sample as a way to normalize each complex activity to mitochondrial number. The following reagents were added to a 1‐cm^2^ cuvette: 100 μM 5,5′‐dithiobis (2‐nitrobenzoic acid) (Sigma‐Aldrich), 1% w/v of the detergent Triton X‐100 to burst cell membranes, 50 μM acetyl coenzyme A, two different volumes of mitochondrial sample (20 μl of mitochondrial sample initially to determine suitable sample volume), the volume was subsequently made up to a final volume of 1 ml with citrate synthase buffer (0.1 mM Tris Hydrochloride in dH_2_0, pH 8.0, warmed to 30°C). After mixing, the cuvette was added to the spectrophotometer to record a baseline, before adding 250 μM of oxaloacetate to begin the reaction and measuring citrate synthase activity (nanomoles of 5‐thio‐2‐nitrobenzoate produced) at 415 nm.

Complex I activity was measured by analyzing the ratio of NADH to ubiquinone oxidoreductase. The following were added to 1‐cm^2^ cuvette: 0.13mM NADH, 2μg/ml Antimycin A, 2μg/ml 1‐trichloromethyl‐1,2,3,4‐tetrahydro‐beta‐carboline (TaClo) or same volume of 100% ethanol vehicle control and two different volumes of mitochondrial sample (20 μl of mitochondrial sample initially to determine suitable sample volume), the volume was subsequently made up to 1 ml with complex I buffer (25 mM potassium phosphate, 5 mM magnesium chloride in dH_2_O, warmed to 30°C). After mixing, the cuvette was added to the spectrophotometer to record a baseline, before adding 65 μM ubiquinone to begin the reaction and measuring complex I activity at 340 nm.

Complex II activity was measured by analyzing the decrease in oxidized electron acceptor 2,6‐dichlorophenolindophenol (DCPIP). The assay was conducted by adding an appropriate volume of complex II buffer containing freshly added 3 mM potassium cyanide (make up to a total reaction volume of 1 ml), 20 mM sodium succinate, and a minimum of two different volumes of mitochondrial sample to a 1.5‐ml Eppendorf tube. The sample was subsequently incubated at 30°C for 10 min to activate mitochondrial complex II, after which the solution was added to a 1‐cm^2^ plastic cuvette, and 3.6 μM antimycin‐A, 50 μM DCPIP, and 5 μM rotenone were added. After mixing, the cuvette was added to the spectrophotometer to record a baseline, before adding 65 μM of ubiquinone to begin the reaction and measuring complex II activity at 600 nm.

Complex IV activity was measured by analyzing the decreasing absorbance at 550 nm associated with reduced cytochrome‐c. The following were added to 1‐cm^2^ cuvette: 0.345 mM n‐dodecyl‐β‐D‐maltoside to solubilize the mitochondrial membrane, 15 μM reduced cytochrome‐c, the volume was subsequently made up to 1 ml with complex IV buffer (20 mM potassium phosphate in dH_2_O, pH 7.4, warmed to 30°C). After mixing, the cuvette was added to the spectrophotometer to record a baseline, before adding a volume of mitochondrial sample (20 μl of mitochondrial sample initially to determine suitable sample volume) and mixing. Measurements were made at 550 nm for 4 min before pausing a second time and adding potassium ferricyanide to oxidize the remaining cytochrome‐c, mixed, and the run was continued until a flat line endpoint was reached.

The levels of citrate synthase activity, complex I activity, complex II activity, and complex IV activity were determined as described previously.^16^

### 2.6 Statistical analysis

One‐way ANOVA corrected for multiple comparisons using the Dunnett's test, and Mann–Whitney *U*‐test were performed to compare conditions based on normality test results (see figure legends).

## 3 RESULTS

**FIGURE 1:**
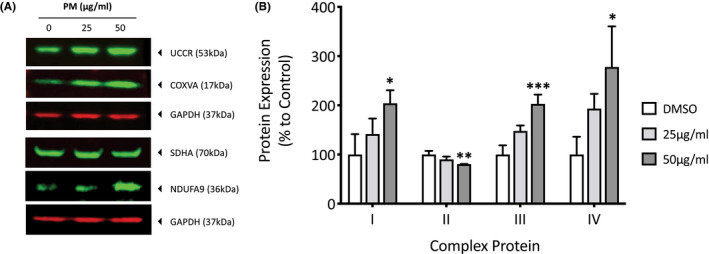
Analysis of the changes in mitochondrial complex protein expression after exposure to particulate matter (PM) in human dermal fibroblasts using western blotting. Dermal fibroblasts were treated with either dimethyl sulfoxide (DMSO), 25 or 50 µg/ml of PM for 7 days before being lysed, and analyzed for the protein expression of mitochondrial complex I (NDUFA9), complex II (SDHA), complex III (UCCR), and complex IV (COXVA) using western blotting (A). Protein expression was normalized to GAPDH using densitometric analysis and presented as mean percentage change + SD to DMSO vehicle control, *n* = 3 (B). Ordinary one‐way ANOVA was performed and corrected for multiple comparisons using Dunnett's test. *p < 0.05, **p < 0.01, ***p < 0.001

Given their roles in oxidative stress and aging, mitochondrial complex protein expression and complex activity was investigated, this will give insight into how air pollutants such as particulate matter affect mitochondrial function and the cellular energy they provide. Dermal fibroblasts were exposed to PM for 7 days before the cells were lysed and analyzed for mitochondrial complex I (NDUFA9), II (SDHA), III (UCCR), and IV (COXVA) proteins using western blotting (Figure 1A). Mitochondrial complex I, III, and IV expression were shown to dose‐dependent increase after exposure to PM, however mitochondrial complex II protein expression seemed to decrease (Figure 1B). Complex I protein, NDUFA9, increased by 41.6% (*p* = 0.29) and 104.1% (*p* = 0.02) after exposure with 25 and 50 μg/ml PM, respectively. Complex II protein, SDHA, decreased by 9.9% (*p* = 0.11) and 19.5% (*p* = 0.008) after exposure with 25 and 50 μg/ml PM, respectively. Complex III protein, ubiquinol cytochrome‐c reductase core protein I (UCCR), increased by 47.8% (*p* = 0.02) and 102.9% (*p* = <0.001) after exposure with 25 and 50 μg/ml PM, respectively. Complex IV protein, COXVA, increased by 93.2% (*p* = 0.14) and 177.8% (*p* = 0.01) after exposure with 25 and 50 μg/ml PM, respectively.

**FIGURE 2:**
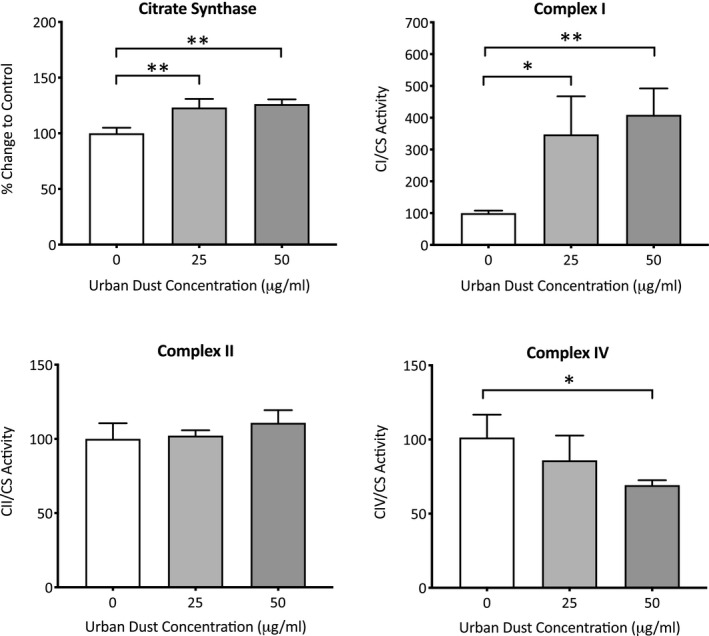
Analysis of particulate matter (PM)‐induced changes in mitochondrial complex activity. Dermal fibroblasts were treated with either dimethyl sulfoxide (DMSO), 25. or 50 µg/ml of PM for 7 days, before the cells were isolated and analyzed for citrate synthase, complex I, complex II, and complex IV activity. Data are expressed as mean percentage change + SD to DMSO vehicle control, *n* = 3. Ordinary one‐way ANOVA was performed and corrected for multiple comparisons using Dunnett's test. *p < 0.05, **p < 0.01

The activities of mitochondrial complexes I, II, and IV of dermal fibroblasts exposed to PM for 7 days were also assessed, with citrate synthase activity used as a normalization factor for mitochondrial mass (Figure 2). Citrate synthase activity showed a dose‐dependent increase of 23.2% (*p* = 0.005) and 26.2% (*p* = 0.003) after exposure to 25 and 50 μg/ml PM, respectively. Similar to NDUFA9 protein expression, complex I activity also showed a concentration‐dependent increases of 247.6% (*p* = 0.02) and 308.9% (*p* = 0.007) after exposure to 25 and 50μg/ml PM, respectively. Despite decreases in mitochondrial complex II protein SDHA, complex II activity remained relatively stable with only minor statistically insignificant increases of 2.2 (*p* = 0.93) and 10.8% (*p* = .25) after exposure to 25 and 50 μg/ml PM, respectively. Finally, complex IV activity showed the greatest difference when compared to the expression of complex IV protein COXVA, showing decreases of 15.4 (*p* = 0.33) and 32.1% (*p* = 0.04) after exposure to 25 and 50 μg/ml PM, respectively, despite increases in protein expression.

**FIGURE 3:**
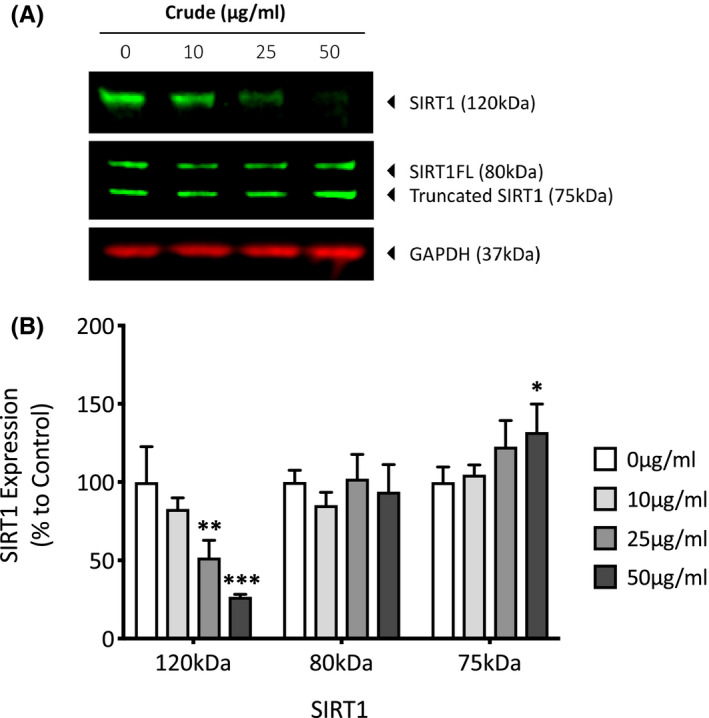
Analysis of sirtuin‐1 (SIRT1) expression in human dermal fibroblasts exposed to particulate matter (PM) for 7 days using western blotting. Dermal fibroblasts were treated with dimethyl sulfoxide (DMSO), 10, 25, or 50 µg/ml of PM for 7 days before being lysed, and analyzed for SIRT1 expression using Western blotting (A). SIRT1 protein was normalized to GAPDH using densitometric analysis, and data are expressed as mean percentage change + SD to the DMSO vehicle control, *n* = 3 (B). One‐way ANOVA was performed and corrected for multiple comparisons using Dunnett's test. *p < 0.05, **p < 0.01, ***p < 0.001

**FIGURE 4:**
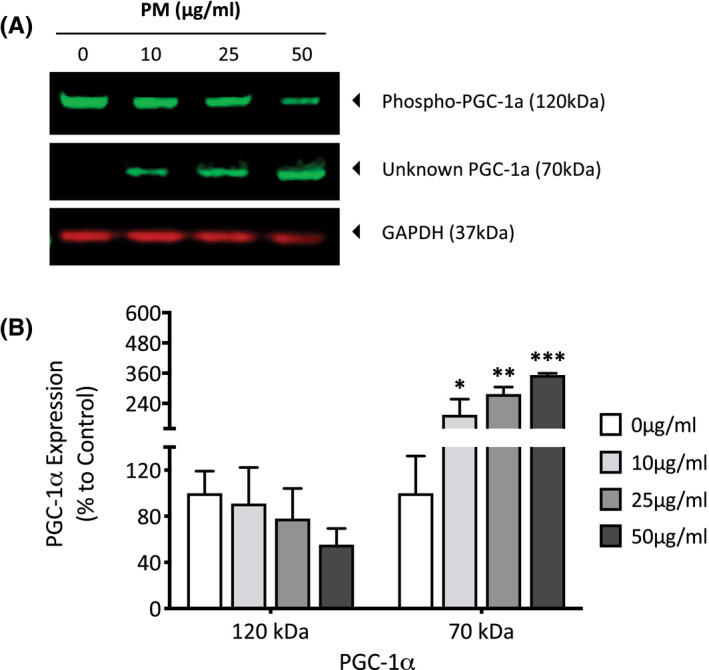
Analysis of peroxisome proliferator‐activated receptor gamma coactivator‐1α (PGC‐1α) expression in human dermal fibroblast after particulate matter (PM) exposure for 7 days using western blotting. Dermal fibroblasts were treated with dimethyl sulfoxide (DMSO), 10, 25, or 50µg/ml of PM for 7 days before being lysed, and analyzed for PGC‐1α expression using western blotting (A). PGC‐1α protein was normalized to GAPDH using densitometric analysis, before presenting as mean percentage change + SD to the DMSO vehicle control, *n* = 3 (B). One‐way ANOVA was performed and corrected for multiple comparisons using Dunnett's test. *p < 0.05, **p < 0.01, ***p < 0.001

PM‐exposed dermal fibroblasts were also analyzed for changes in SIRT1 and PGC‐1α expression using western blotting to assess any alterations in mitochondrial biogenesis and energy metabolism in response to air pollution. SIRT1 is a known prosurvival protein, involved in the promotion of mitochondrial biogenesis via PGC‐1α and can also stimulate the expression of antioxidants such as SOD2 to help neutralize increases in ROS.^17^ The SIRT1 antibody recognized three forms of SIRT1: the predicted 120‐kDa SIRT1 (the actual molecular weight of SIRT1 is 81 kDa; however, the protein tends to appear higher due to post‐translational modifications), the 81‐kDa form (SIRTFL), and a C‐terminally truncated 75‐kDa form (75SirT1) that is generated after proteolysis of full‐length SIRT1 (Figure 3A). Western blot analysis of SIRT1 showed a concentration‐dependent decrease of 17.2 (*p* = 0.32), 48.2 (*p* = 0.005), and 73.4% (*p* = <0.001) for 10, 25, and 50 µg/ml, respectively. There was also a complimentary increase in the C‐terminally truncated 75‐kDa isoform of 4.8 (*p* = 0.95), 22.6 (*p* = 0.17), and 31.9% (*p* = 0.05) for 10, 25, and 50 µg/ml, respectively (Figure 3B). There were no significant changes in SIRTFL isoform after exposure to PM. PGC‐1α is a key protein in modulating both mitochondrial biogenesis and function through transcription regulation. The antibody recognized two forms of PGC‐1α: the predicted 120‐kDa phopshorylated‐PGC‐1α at the Ser571 site and an unknown 70‐kDa form (Figure 4A). Due to the diverse nature of this protein and its ability to localize in different organelles, it exists as multiple isoforms and is capable of a number of post‐translational modifications. It is therefore difficult to state with certainty what post‐translational modification PGC‐1α has undergone to produce this 70‐kDa form, however due to its large number of acetyl groups and the observed alteration in SIRT1 protein expression it is possible that this is deacetylated PGC‐1α. Phospho‐PGC‐1α showed a concentration‐dependent decrease of 9.1 (*p* = 0.93), 22 (*p* = 0.56), and 44.6% (*p* = 0.12), and there was also a complimentary increase in the 70‐kDa form of 95.3 (*p* = 0.04), 177.1 (*p* = 0.001), and 252.5% (*p* = <0.001) for 10, 25, and 50 µg/ml, respectively (Figure 4B).

**FIGURE 5:**
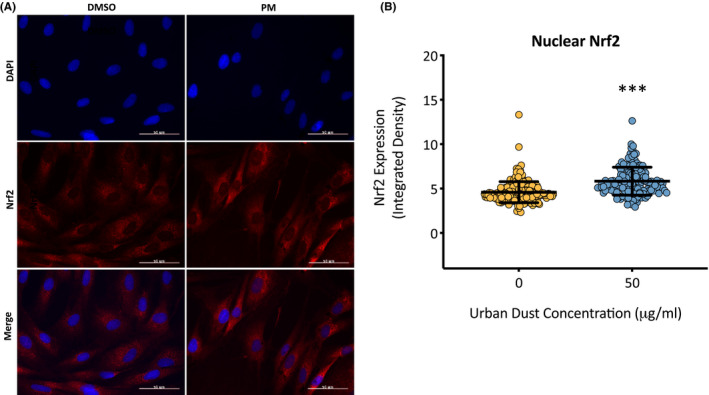
Analysis of cellular distribution of antioxidant transcription regulator Nrf2 after particulate matter (PM) exposure in human dermal fibroblasts using immunocytochemistry. Dermal fibroblasts were treated with dimethyl sulfoxide (DMSO) or 50 µg/ml of PM for 24 h and fixed. Cells were then stained with anti‐Nrf2 antibody and counterstained with 4,6‐diamidino‐2‐ phenylindole (DAPI), before being visualized using fluorescence microscopy (A). Extent of nuclear Nrf2 translocation was assessed, with around 200–300 cells analyzed for each treatment group (B). Mann–Whitney *U*‐test was performed to compare conditions, ***p < 0.001

**FIGURE 6:**
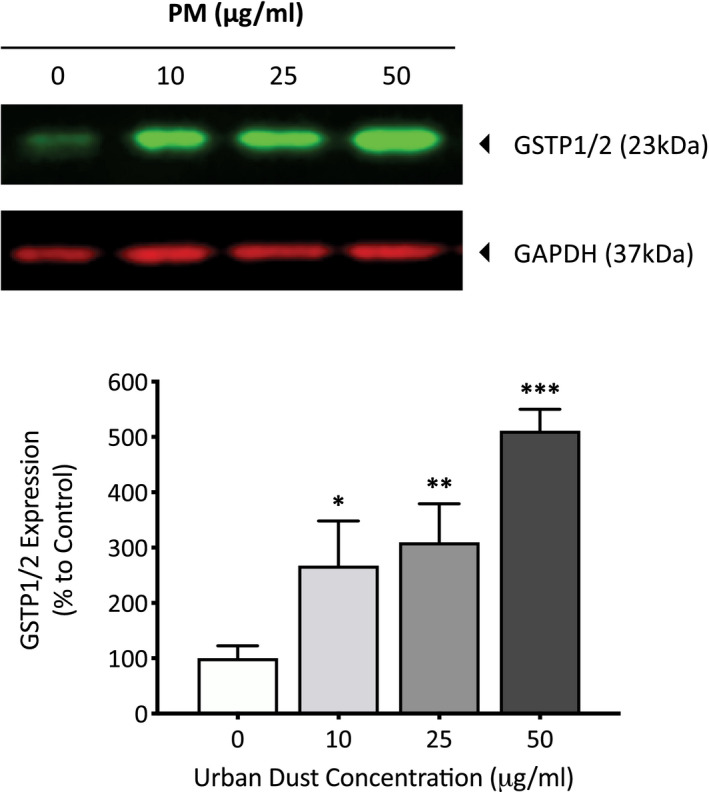
Analysis of changes in expression of cytosolic antioxidant enzyme GSTP1/2 after PM exposure in human dermal fibroblasts using western blotting. Dermal fibroblasts were treated with dimethyl sulfoxide (DMSO), 10, 25, or 50 µg/ml of PM for 7 days before being lysed, and analyzed for cytosolic antioxidant enzyme GSTP1/2 expression using western blotting. Protein expression was normalized to GAPDH using densitometric analysis and presented as mean percentage change + SD to DMSO vehicle control, *n* = 3. Ordinary one‐way ANOVA was performed and corrected for multiple comparisons using Dunnett's test. *p < 0.05, **p < 0.01, ***p < 0.001

**FIGURE 7:**
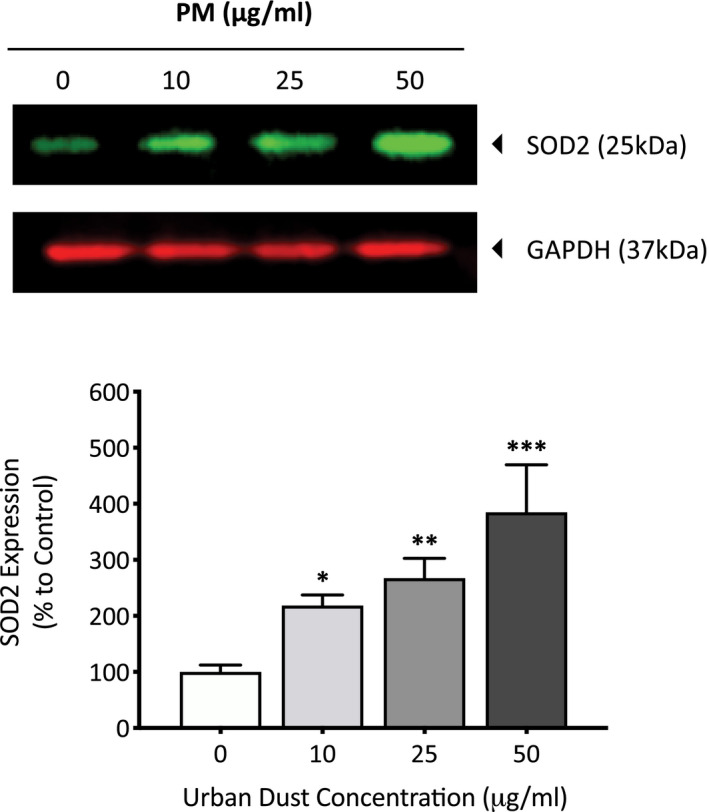
Analysis of changes in expression of mitochondrial antioxidant enzyme SOD2 after particulate matter (PM) exposure in human dermal fibroblasts using western blotting. Dermal fibroblasts were treated with dimethyl sulfoxide (DMSO), 10, 25, or 50 µg/ml of PM for 7 days before being lysed, and analyzed for mitochondrial antioxidant enzyme SOD2 expression using western blotting. Protein expression was normalized to GAPDH using densitometric analysis and presented as mean percentage change + SD to DMSO vehicle control, *n* = 3. Ordinary one‐way ANOVA was performed and corrected for multiple comparisons using Dunnett's test. *p < 0.05, **p < 0.01, ***p < 0.001

Nrf2 is a protein involved in the transcription regulation of both the basal and induced expression of antioxidant proteins to protect against oxidative damage. Under oxidative stress oxidants dissociate KEAP1 from Nrf2 allowing it to translocate to the nucleus and regulate the transcription of key proteins involved in antioxidant defenses. To determine the effect of PM on the activation of Nrf2, primary dermal fibroblasts were exposed to 50 µg/ml of PM for 24 h before the cells were fixed and the extent of nuclear translocation of Nrf2 was analyzed using immunocytochemistry (Figure 5A). Cells exposed to PM experienced a 27.1% (*p* = <0.001) increase in nuclear Nrf2 compared to the DMSO vehicle control (Figure 5B). Active Nrf2 regulates the expression of antioxidant proteins which help combat increases in ROS, examples include mitochondrial antioxidant SOD2 and cytosolic antioxidant GSTP1/2. Since PM caused an increase in nuclear translocation of Nrf2, it was of interest to determine whether Nrf2‐regulated antioxidant proteins were also increased and to what extent. Primary dermal fibroblasts were exposed to varying concentrations of PM for 7 days, before the cells were lysed and assessed for expression of SOD2 and GSTP1/2 proteins using western blotting. PM induced a concentration‐dependent increase in cytosolic antioxidant GSTP1/2 expression in dermal fibroblasts, with increases of 167.5 (*p* = 0.02), 209.6 (*p* = 0.005), and 411.1 (*p* = <0.001) for 10, 25, and, 50 µg/ml PM, respectively (Figure 6). PM also induced a concentration‐dependent increase in mitochondrial antioxidant SOD2 expression in dermal fibroblasts, with increases of 118.5 (*p* = 0.04), 167.3 (*p* = 0.006), and 284.8 (*p* = <0.001) for 10, 25, and 50 µg/ml PM, respectively (Figure 7).

## 4 DISCUSSION

In this study, the PM “urban dust” used was a compound mixture prepared from atmospheric particulate matter in the Washington, DC area. This mixture generally typifies PM in ambient air pollution from an urban area, and contains polycyclic aromatic hydrocarbons (PAHs) and quinones, amongst other compounds.^18^ Previous work has shown this PM to induce the activation of the aryl‐hydrocarbon receptor which is responsible for transcription regulation of phase I xenobiotic metabolizing enzymes (such as CYP1A1).^19^ These enzymes can convert PAHs to additional quinones which are capable of generating ROS through redox cycling, and if not neutralized can cause oxidative damage.^20^ With the scarcity of published research specifically investigating the adaptive responses of human skin cells after prolonged exposure to air pollutants such as PM, it was therefore of interest to investigate these cellular responses. This may provide better insight into which systems need support when developing interventions to protect individuals against air pollution‐induced damage.

With previous work and work published by other groups indicating that the damage induced by PM is orchestrated by ROS, changes in mitochondrial activity and complex protein expression were relevant targets to assess. An increase in mitochondrial complex I, III, and IV protein subunits but not complex II, could mean that complex II is not as well adapted as the other complexes to continuous exposure to oxidative stressors. This could be because unlike other complexes that have a larger number of both mtDNA and nDNA encoded subunits, complex II has only four nDNA encoded subunits. This could mean oxidative stress‐induced changes in nDNA encoded protein expression may be more pronounced in complex II, as unlike the other complexes with more subunits there are not enough additional subunits to compensate for any decreased subunit expression. It has also been speculated that the reason for complex II being entirely nuclear‐encoded (and therefore within the protection of the nuclear‐repair mechanisms instead of the mtDNA which is under high ROS insult), is because complex II dysfunction is so detrimental that protective measures to prevent its dysfunction have been implemented by cells.^21^ Oxidative stress‐induced decreases in complex II protein could also explain why there has been an observed decrease in complex II protein with age reported, as increased oxidative stress seems to be a characteristic of aging.^22^ A decrease in complex II expression may actually act as an adaptation to unfavorable conditions, with one study showing that hypoxic conditions induce decreases in complex II activity but increases in complex I, III, and IV activity, and this results in a >60% reduction in superoxide leakage from the ETC.^23^ This could be explained by mitochondrial complexes forming super‐complexes comprising of either I/III or I/III/IV, as complex II is not part of the assembly but instead left to free‐float in the mitochondria. Although the role of super‐complex formation is still being elucidated, it is thought to stabilize complex I, in an effort to limit oxidative damage by sequestering susceptible sites.^24^ This could explain the decline in complex II protein expression, as SDHA may not be required in the newly formed super‐complex. The concentration‐dependent increase in complex I activity after PM exposure does compliment the theory of super‐complex formation as a way to stabilize complex I during its increased activity, however the other PM‐induced changes in the other complex activities do raise further questions. The nonsignificant changes in complex II activity despite decreases in SDHA protein expression could be explained by the small decreases in protein expression not being enough to drastically alter the complex activity, or that this subunit it not the most essential when it comes to complex II‐linked respiration.^25^ The PM‐induced changes in complex IV activity are especially of interest, because despite a substantial concentration‐dependent upregulation in COXVA protein expression, there was a contradictory concentration‐dependent decrease in complex IV activity. This would indicate that either complex IV activity is being inhibited, the increased subunits are dysfunctional (possibly due to oxidative damage) or not required for complex IV activity. With complex IV seemingly integral to the proper formation and stabilization of complex I in super‐complexes, it is possible that the protein itself is the stabilizing factor and that the complex IV activity is not necessary.^24^, ^26^

One of the first lines of cellular defense against oxidative damage, is to induce the expression of antioxidant enzymes to neutralize electrophilic substances and ROS. This is initiated by the activation of Nrf2, which can be observed in the cell by the shuttling of this transcription factor from the cytoplasm to the nucleus. The increased complex I activity also has a paradoxical role, not only increasing the generation of superoxide ions, but also activating antioxidant response through Nrf2 to eliminate them.^27^ As seen in this study, PM exposure induced an increase of 27.1% in nuclear Nrf2 compared to the DMSO vehicle control, confirming the presence of oxidative stress and therefore oxidants within the PM mixture. With Nrf2 activated, it was a question as to what extent the protein regulated the transcription and subsequently the translation of key antioxidant defense proteins such as GSTP1/2 and SOD2.^28^ GSTP1/2 is involved in conjugating quinones and ROS with glutathione to help reduce any oxidative damage from these molecules.^29^ Although GSTP1/2 was long thought of to be a cytosolic antioxidant enzyme, it has now been suggested that this enzyme can also localize in the nucleus and mitochondria of cells in order to combat any increased oxidative stress.^30^ With dose‐dependent increases in GSTP1/2 expression after exposure to PM, it seems the cell is able to adapt to the increased electrophile/ROS presence by increasing its expression of GSTP1/2. However, GSTP1/2 only serves a function as long as there is a source of glutathione to catalyze the conjugation of electrophiles/ROS, therefore continuous exposure to stressors that induce ROS generation such as UV and air pollution will eventually deplete glutathione stores and allow ROS to damage cellular components if the stores are not replenished.^31^, ^32^ Oxidative damage has now been implicated in various skin disorders, including premature aging, psoriasis, and atopic dermatitis and if oxidative stress increases GSTP1/2 expression it may explain the decrease in glutathione in these conditions.^33^, ^34^, ^35^ SOD2 transforms superoxide ROS produced from the ETC into hydrogen peroxide and diatomic oxygen, therefore plays an important role in protecting the cell from ROS originating in the mitochondria. The dose‐dependent increase in SOD2 after PM exposure indicates the increased presence of superoxide ROS that need to be neutralized, which has also been observed in UV‐irradiated and senescent dermal fibroblasts.^36^, ^37^, ^38^, ^39^ This increase in superoxide is most likely a result of the increased complex I activity which results in an increased leakage of superoxide anions. The increased SOD2 will convert superoxide anions into hydrogen peroxide, which although supposedly less harmful, hydrogen peroxide has also been shown to induce some of the hallmark changes seen in aging skin. This includes increases in MMP‐1 expression, capable of degrading major structural skin components like collagen‐I.^40^ It is a fine balance between adaptive protection against increases in ROS and these same systems being overwhelmed by an excess of ROS, and oxidative damage within the cell can then ensue.

With the observed spike in complex I activity after 7 days of PM exposure, it was also of interest to investigate whether the increased NAD+/NADH ratio was adequate enough to activate SIRT1 which uses NAD+ as a substrate. A concentration‐dependent decrease in full‐ length SIRT1 expression and a complimenting increase in the 75‐kDa truncated form indicates that PM is in fact modulating SIRT1 to some extent; however, the link between truncation and SIRT1 function is not well researched. It is possible that the decrease in full‐length SIRT1 is actually due to it becoming modified into an active form, or that chronic SIRT1 activation ultimately marks the protein for proteasomal degradation.^9^ Most studies investigating the truncated form of SIRT1 conclude that it is a prosurvival modification of sorts, with it being shown to localize in the mitochondrial membrane and associate with cytochrome‐c, consequently inhibiting its involvement in apoptosis.^41^ This may explain why a decrease in full‐length SIRT1 and an increase in this truncated form has been shown to be enhanced in aging tissue, most likely in an attempt to extend cell survival during times of cellular stress.^42^ With PM being shown to induce ROS generation, this could induce lysosomal cathepsin B release, allowing it to truncate SIRT1 and initiate downstream pathways in order to protect itself from oxidative stress.^43^ One such downstream pathway is the deacetylation of PGC‐1α which allows it to translocate to the nucleus and regulate transcription of genes associated with the upregulation of mitochondrial biogenesis and energy metabolism.^44^ Because phosphorylation of PGC‐1α is a prerequisite to deacetylation by SIRT1, phosphorylated PGC‐1α was analyzed. PM induced a concentration‐dependent decrease (although not statistically significant) in phosphorylated PGC‐1α, and a concentration‐dependent increase in an unknown form at 70 kDa. Given the antibody recognizes PGC‐1α that has been phosphorylated at the S571 site, that deacetylation follows phosphorylation, and that SIRT1 has been altered, this band could represent deacetylated phosphorylated PGC‐1α. If this is the case, then this would allow the protein to regulate transcription of mitochondrial proteins, which would explain the increased protein expression of complex subunits NDUFA9, UCCR, and COXVA.

Unlike the prevailing theory of a gradual decline in cellular functions with age, the data presented in this paper could corroborate a more biphasic model of aging, where cells adaptively increase their metabolism from young to middle age in order to deal with the increased need for cellular defenses, before subsequently reducing in later stages of life.^45^ This could be linked to the induction of cell senescence after oxidative stress which has been shown to increase mitochondrial respiration, most likely to fuel the synthesis and secretion of senescence‐associated proteins.^46^, ^47^ Transcriptomic analysis of mitochondrial complex subunits from the dermis of subjects of different ages also shows an initial upregulation of the majority of genes between ages 20 and 50, followed by a downregulation thereafter.^48^ The same paper show that complexes encoded by both nDNA and mtDNA are more responsive to protective intervention than the likes of complex II which is only encoded by nDNA. This was hypothesized to be a result of compensatory action of the other complexes, or that mitophagy rescues complex II, masking the real changes in expression and activity. Similarly, this could explain why SOD2 activity has been shown to increase up to the age of 60 years old and decrease thereafter in dermal fibroblasts, which may act as an adaptive protection mechanism to reduce superoxide anion damage in aging cells.^37^ Whether induced aging follows the same biphasic model as chronological aging has yet to be elucidated. This study could confirm the biphasic theory in induced aging and also that mitochondrial complex II is the most susceptible to the oxidative damage associated with aging of all mitochondrial complexes.

## CONFLICT OF INTEREST

The author declares no conflict of interest.

## AUTHOR CONTRIBUTIONS

Mark A. Birch‐Machin as the senior and corresponding author co‐designed the research project with Bhaven Chavan. Wil J. Reynolds and Amy Bowman performed the experiments and Wil J. Reynolds wrote the paper. Adam Critchley and Ben Griffiths provided the tissue from which the cells were isolated. Mark A. Birch‐Machin, Bhaven Chavan, and Peter S. Hanson for comments on the manuscript.
